# Three-dimensional correction of cubitus varus deformity using patient-specific 3D-printed osteotomy guides

**DOI:** 10.3389/fsurg.2026.1804208

**Published:** 2026-06-02

**Authors:** Mei-Ren Zhang, Xiao Zeng, Jiang-Long Guo, Kui Zhao, Jian-Hui Hu, Jian-Hao Guan

**Affiliations:** 1 Guangdong Provincial Hospital of Chinese Medicine, ZhuHai Hospital, Orthopedics Trauma Department, ZhuHai, Guangdong, China; 2 Guangzhou University of Chinese Medicine Second Clinical College, Guangzhou, Guangdong, China

**Keywords:** accurate osteotomy, cubitus varus deformity, custom-matchedsurgical osteotomy template, reduction templates, three-dimensional printing

## Abstract

**Background:**

Various three-dimensional (3D) corrective osteotomy techniques have been reported for the treatment of cubitus varus deformity. However, achieving accurate correction through a limited lateral incision remains technically challenging. This study introduces a method for accurate 3D osteotomy of cubitus varus deformity using a limited lateral incision.

**Methods:**

Five patients (2 males and 3 females) with cubitus varus deformity following supracondylar fracture underwent 3D corrective osteotomy using 3D-printed, patient-specific osteotomy templates, along with custom location and reduction guides, between August 2022 and January 2025. These cases were evaluated retrospectively. Clinical outcomes assessed included pre- and postoperative carrying angles, operative time, elbow joint function, intraoperative blood loss, degree of osteotomy, time to bone union, and postoperative complications.

**Results:**

The mean carrying angle on the affected side improved significantly from −15.74° ± 6.58° (varus) preoperatively to 7.77° ± 3.94° (valgus) postoperatively. The mean tilting angle improved from 54.8° ± 7.40° to 51.4° ± 2.33°. Elbow range of motion normalized in all patients, with a mean increase in flexion angle of 24° ± 8° (range: 15°–35°). Hyperextension of the elbow and internal rotation of the shoulder were also corrected. Bone union was achieved at a mean of 2.6 ± 0.49 months (range: 2–3 months). The average operative time was 139.6 ± 22.26 min (range: 116–175 min), and mean intraoperative blood loss was 42 ± 31.87 mL (range: 10–100 mL). The mean correction angle achieved through osteotomy was 23.51° ± 8.79° (range: 12.43°–33.43°). According to the Mayo Elbow Performance Index (MEPI), all five patients achieved excellent outcomes at the final follow-up (mean: 21.6 ± 4.8 months), with no reports of poor results, recurrence of varus deformity, or wound-related complications. One patient exhibited transient ulnar nerve symptoms postoperatively. No patients reported prominence of the lateral humerus.

**Conclusion:**

The use of a 3D-printed, patient-specific osteotomy guide combined with custom location and reduction templates enables safe, accurate, and reproducible 3D correction of cubitus varus deformity through a small lateral incision. This surgical technique, grounded in 3D computer simulation, reduces variability between surgeons and may represent a viable therapeutic option for the correction of cubitus varus deformity.

## Introduction

Cubitus varus deformity is a common complication following a supracondylar fracture of the humerus (1–4), is characterized by a complex three-dimensional (3D) malalignment. This deformity is quantitatively assessed through key radiographic angles: the carrying angle (CA) and the tilting angle (TA). The CA, measured on an anteroposterior radiograph, is the angle between the longitudinal axes of the humerus and ulna in full elbow extension; a negative value indicates varus alignment. The TA, measured on a true lateral radiograph, represents the sagittal plane alignment as the angle between the humeral shaft axis and the metaphyseal axis of the distal humerus, with an increased value typically indicating a hyperextension deformity. Accurate correction of all three deformity components is essential to prevent long-term sequelae, including limited elbow flexion, joint instability, tardy ulnar nerve palsy, and to achieve satisfactory cosmetic results (5–12).

Although various surgical methods have been proposed to correct cubitus varus deformity (13–16), achieving accurate correction of this complex, three-dimensional (3D) deformity remains technically challenging. The condition involves deformity in all three planes: varus angulation in the coronal plane, hyperextension in the sagittal plane, and internal rotation in the horizontal plane. As such, triplanar osteotomy techniques have been recommended to achieve comprehensive 3D correction (16, 17), despite their associated technical demands and surgical risks (18–23).

Recent advances in computer simulation and the development of custom-matched surgical guides and implants using 3D printing have addressed many of these challenges, enabling more accurate, straightforward, and safer 3D corrections (16, 19, 23–25). However, differences in surgical incisions and fixation methods remain across techniques. In this study, we introduce a a laterally approached, limited-incision (8 cm) 3D corrective osteotomy for cubitus varus deformity, utilizing preoperative computer simulation and custom 3D-printed surgical templates. We also present clinical and radiographic outcomes of this approach.

## Materials and methods

### Subjects

Between August 2022 and June 2025, five consecutive patients with cubitus varus deformity (Table 1)—resulting from malunion of a distal humerus supracondylar fracture—underwent 3D corrective osteotomy using 3D-printed, patient-matched surgical osteotomy guides, along with custom location and reduction templates. The procedure was performed through a limited lateral incision and was based on preoperative computer simulation using 3D CT models. During this period, no patients were excluded, as all eligible patients provided informed consent and met the inclusion criteria. The inclusion criteria were: (1) a diagnosis of cubitus varus deformity secondary to malunited distal humerus fracture, and (2) patient-reported dissatisfaction with cosmetic appearance or functional limitations. There was no minimum follow-up period mandated for inclusion in this initial technical feasibility study; however, the actual follow-up duration for each patient is detailed in Table 1 and ranged from 6 to 35 months.

**Table 1:** Details of Patients Undergoing 3D Corrective Osteotomy for Cubitus Varus Deformity.

Patient No./ Sex/Age (y)	Age at initial injury (y)	Side	Mechanism of injury	Time from injury to surgery (mo)	Follow-up (mo)	MEPI score	Operation time (min)	Intraoperative blood loss (mL)	Increased ﬂexion angle (°)	Osteotomy degrees (°)	Osteotomy end union time (mo)	Postoperative complications
1/F/12	11	L	Fall	8	35	100	116	20	30	34.33	3	No
2/M/37	9	L	Fall	336	29	100	175	100	35	33.40	2	No
3/F/7	3	R	Fall	48	24	100	152	10	15	12.42	2	No
4/F/11	5	R	Fall	72	12	100	117	30	15	17.69	3	Ulnar nerves injury^a^
5/M/44	9	L	Fall	420	6	100	138	50	25	19.71	3	No
Mean ± SD	7.4			176.8 ± 167.7	21.6 ± 4.8		139.6 ± 22.26	42 ± 31.87	24 ± 8	23.51 ± 8.79	2.6 ± 0.49	

a Transient ulnar nerve symptoms, fully recovered after wire removal and neurotrophic treatment.

The mean age at the time of initial injury was 7.4 ± 2.94 years (range: 3–11 years). All five patients (2 males and 3 females) underwent postoperative x-ray examination and were evaluated retrospectively, with a mean follow-up period of 21.1 ± 10.7 months (range: 6–35 months). The mean age at the time of corrective osteotomy was 22.2 ± 15.2 years (range: 7–44 years), and the mean duration from injury to surgery was 177 months (range: 8–420 months). Four of the five patients had been treated with cast immobilization for the initial fracture, while one had undergone open reduction and internal fixation with Kirschner wires. All patients presented with unilateral elbow deformity and reported dissatisfaction with the cosmetic appearance of the affected limb. In addition to aesthetic concerns, three patients (Cases 1, 2, and 5) experienced restricted elbow flexion exceeding 20° compared with the unaffected side. No patient reported hyperextension of the elbow.

### Image acquisition and preoperative simulation

#### Step 1: 3D evaluation of cubitus varus deformity

**Figure 1:**
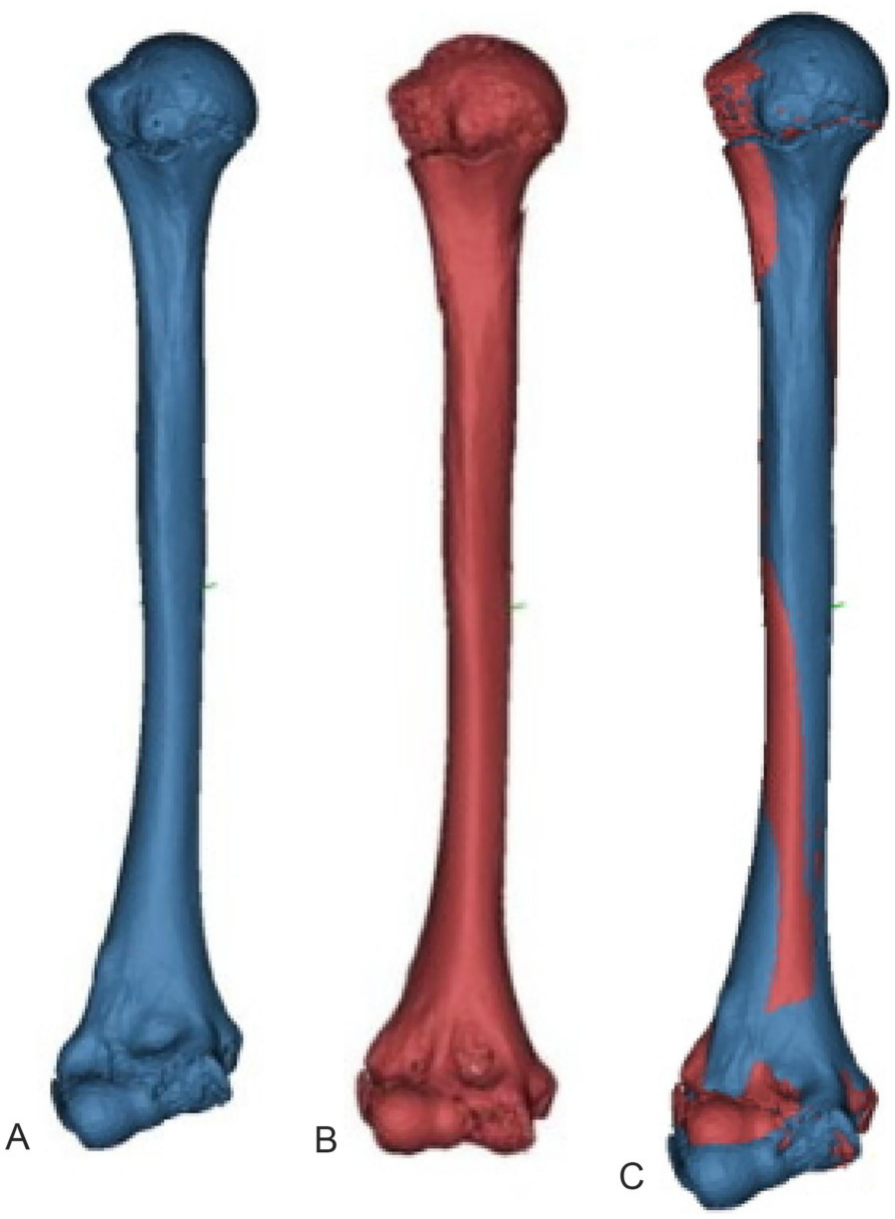
**(A)** Affected side of humerus (dark blue); **(B)** the mirror image of the contralateral normal humerus (dark red); **(C)** the proximal site of the affected humerus was superimposed onto the mirror image of the corresponding part on the contralateral, normal side;.

Computerized Tomography (CT) data of both humeri were obtained, and corresponding 3D bone models were created. Scanning was performed with the forearms in maximum supination using a low-radiation dose protocol on a CT scanner (General Electric LightSpeed-640, GE, Milwaukee, WI) with a slice thickness of 0.5 mm. Three-dimensional surface models of both humeri were generated from the imaging data, and the deformity was evaluated by comparing the affected humerus with the mirrored image of the contralateral, normal side using 3D modeling software (Magics RP; Materialise, Leuven, Belgium) (Figures 1A,B).The proximal part of the affected humerus was superimposed onto the mirrored image of the corresponding area on the normal side (proximal registration) (Figure 1C). The same process was applied to the distal humerus (distal registration). The software calculated the 3D magnitude of deformity automatically by analyzing the transformation data required to align both the proximal and distal segments.

#### Step 2: planning the 3D corrective osteotomy

**Figure 2:**
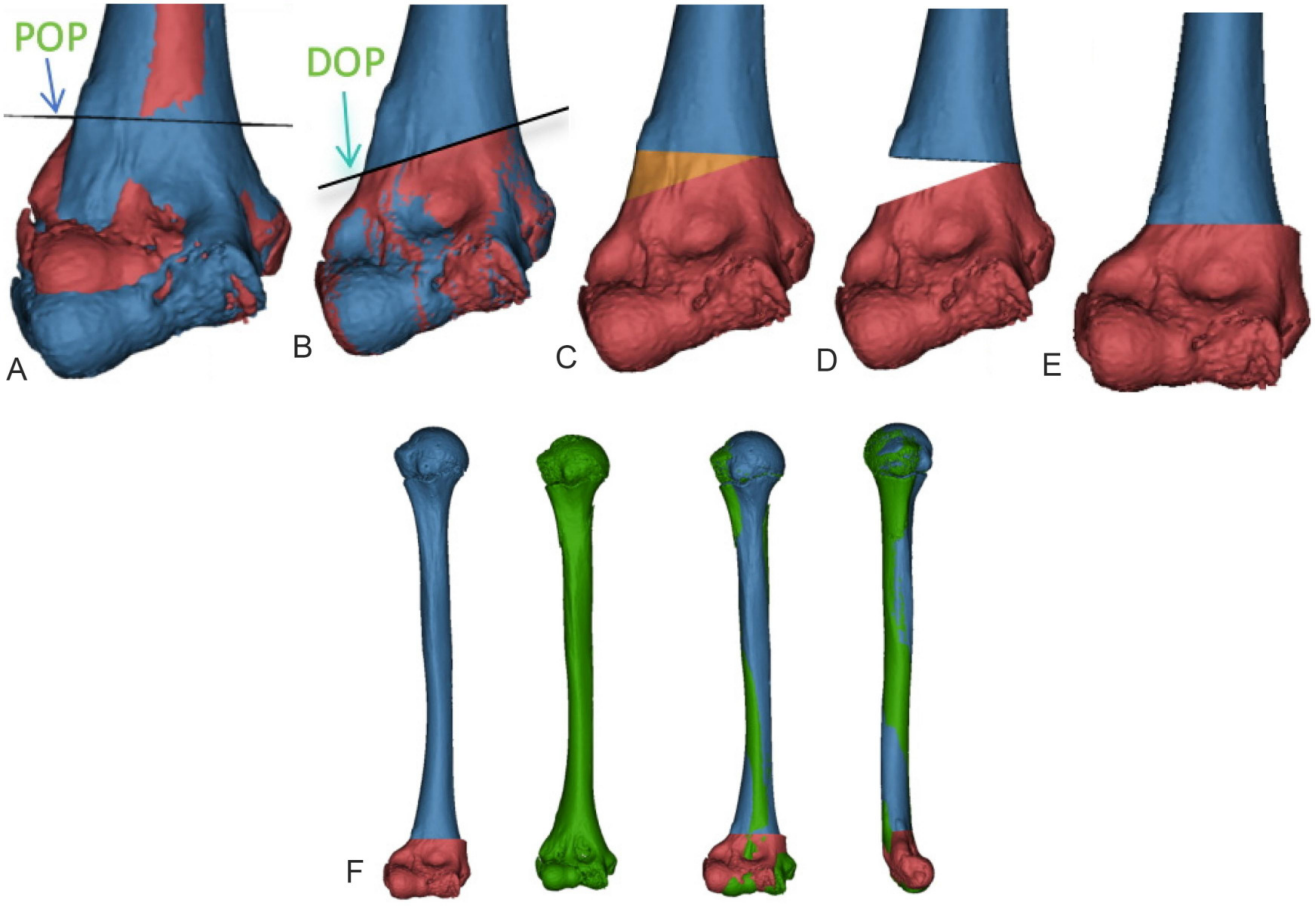
**(A)** the proximal osteotomy plane (POP)roughly vertical to humerus axis and proximal about 0-1 cm to the olecranon fossa, make the first osteotomy surface, name and save the plane as the proximal osteotomy plane (POP); **(B)** mirror distal part of contralateral, normal side after osteotomy by the proximal osteotomy plane was superimposed onto affected side with the distal articular surface completely fitting by moving and rotating, the distal osteotomy plane (DOP) was determined by moving the proximal osteotomy plane (POP) by the correction amount. **(C,D)** The wedge shaped segment (orange bone) between the distal and proximal osteotomy planes was removed from the affected humerus. **(E)** Complete the simulation of 3-D correction by moving the distal segment of the humerus to the proximal segment of the humerus. **(F)** Verify that the overall appearance of the extremity has been appropriately corrected on the computer monitor.

A simulated 3D corrective lateral closing corrective osteotomy was performed based on the deformity analysis. Two osteotomy planes were created using the software: the proximal osteotomy plane (POP) and the distal osteotomy plane (DOP). The POP was defined by positioning a virtual plane perpendicular to the humeral axis, approximately 0–1 cm proximal to the olecranon fossa on the mirrored image of the normal side. This plane was saved as the POP (Figure 2A). The DOP was then positioned parallel to the articular surface of the distal humerus, located approximately 1–2 cm proximal to the trochlea-capitellum junction, corresponding to the preoperatively calculated correction angle (Figure 2B). The wedge-shaped segment between the POP and DOP was digitally removed using the software's edit function (Figures 2C,D). To complete the correction simulation, the distal segment of the affected humerus was repositioned to meet the proximal segment (Figure 2E), and the final alignment was visually verified to confirm the restored anatomy (Figure 2F).

#### Step 3: design of patient-specific surgical templates

1. Design of the Custom-Made Surgical Reduction Template

The wedge-shaped segment between the distal and proximal osteotomy planes (DOP and POP) was removed from the affected humerus. The simulation of 3D correction was completed by repositioning the distal humerus segment to align with the proximal segment. A custom reduction template was designed to fit the posterolateral surface of the corrected distal humerus.

**Figure 3:**
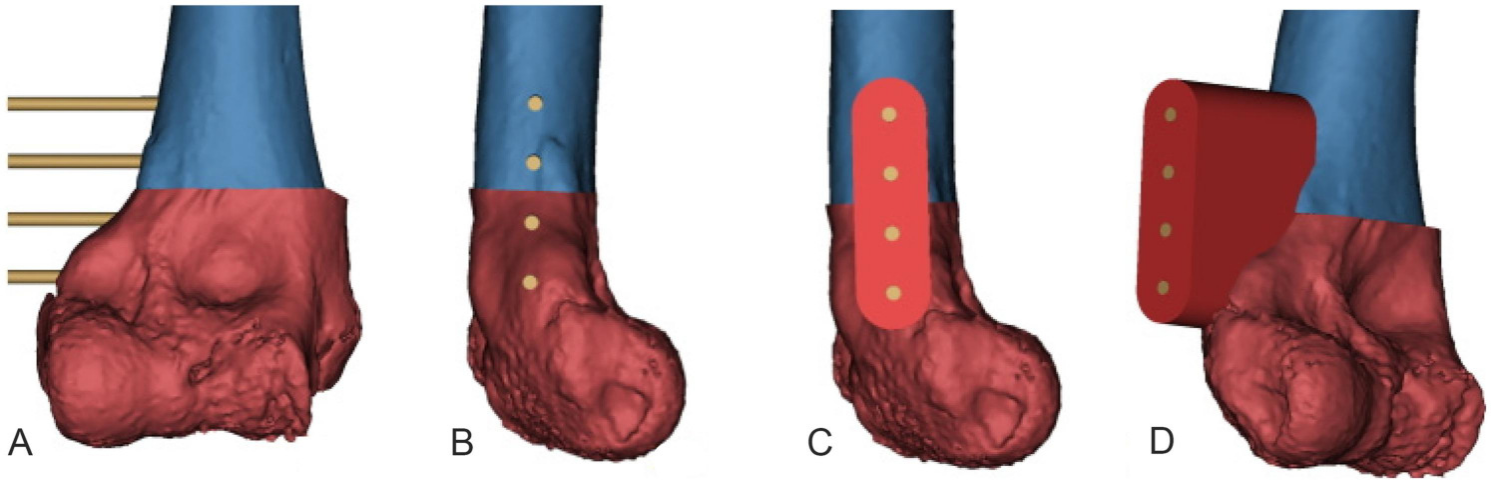
Design custom-made surgical reduction guides **(A,B)** four guide pins were setting placed parallel in the middle line of the lateral distal humerus while vertical to humerus axis with interval 8 mm each other. The midpoint of the second and third guide pin were setting on the osteotomy surface; **(C,D)**.custom-made surgical reduction guides were design achievement based on the 4 guide pins and their positions while completely matching lateral side the distal humerus by solidworks software.

Four guide pins were positioned parallel to each other along the midline of the lateral distal humerus, perpendicular to the humeral axis, with 8 mm spacing and a diameter of 2.0 or 2.5 mm (Figure 3A). The midpoint between the second and third guide pins was placed on the osteotomy surface (Figure 3B). Pin spacing and diameter could be adjusted based on the patient's bone size. The surgical reduction template was then designed using SolidWorks software based on the placement and orientation of the four guide pins (Figures 3C,D). This template was used to assist with reduction and temporary fixation during surgery.

2. Design of the Custom-Made Surgical Osteotomy Template

**Figure 4:**
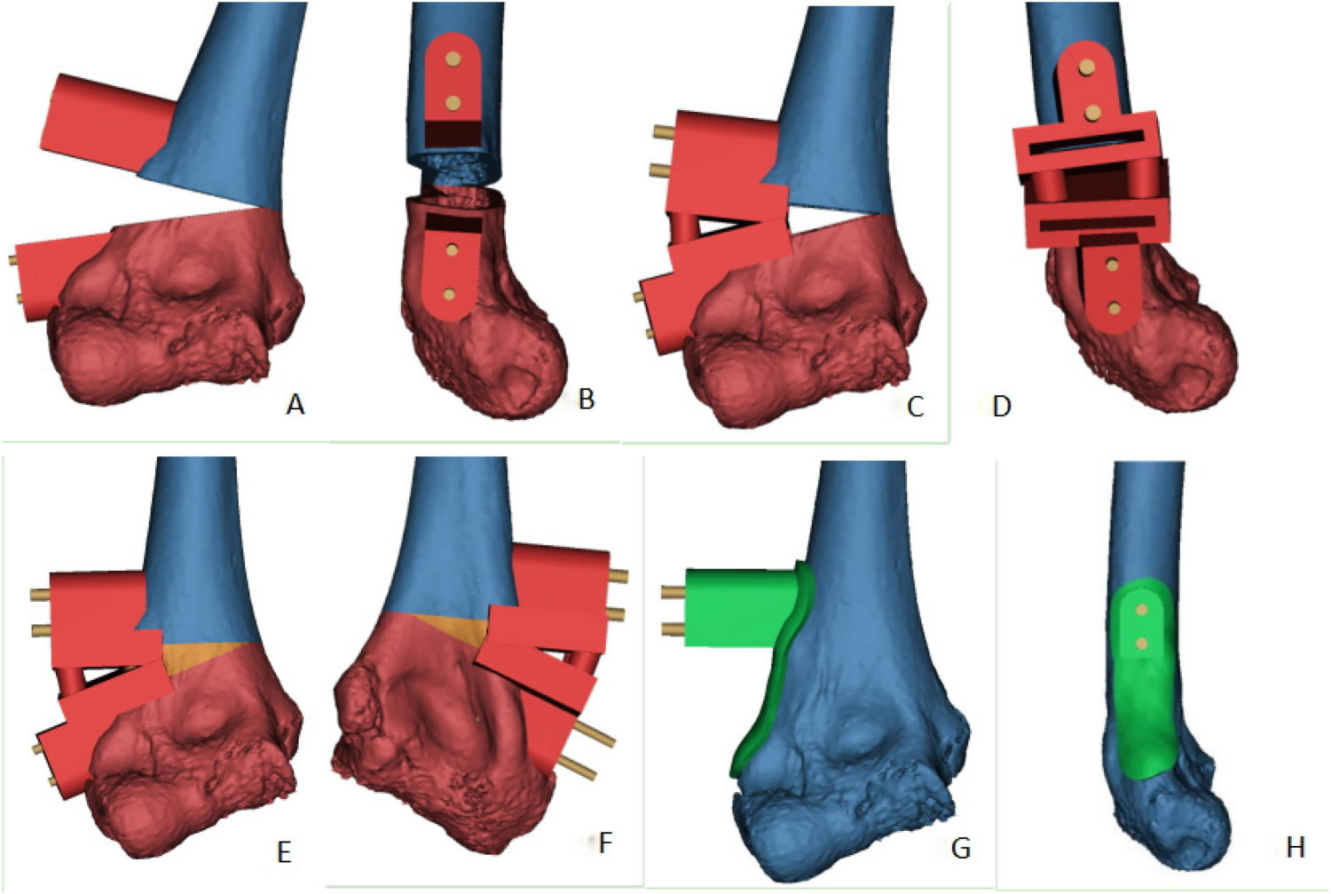
Design custom-made surgical osteotomy guide and location guide. **(A,B)** custom-made surgical reduction template was separated with upper and lower parts on osteotomy plane and together with the distal humerus back to its original uncorrective place by solidworks software. **(C,D)** Two osteotomy slits were designed through the proximal osteotomy plane(POP) and the distal osteotomy plane(DOP) and connected with the upper and lower separated surgical reduction template respectively by a connecting rod to increase the stability of surgical osteotomy template. **(E,F)** surgical osteotomy template were accomplish after matching the lateral side the distal humerus by solidworks software. **(G,H)**. A positioning template was designed based on two guide pins upper to osteotomy plane after matching the lateral side of the distal humerus by solidworks software.

The surgical reduction template was divided into upper and lower components along the proximal osteotomy plane (POP) using the software. The distal humerus of the affected side was then returned to its original uncorrected position (Figures 4A,B). Two osteotomy slits were designed through the POP and DOP, each connected to the corresponding section of the reduction guide by a connecting rod to enhance template stability (Figures 4C,D). The final osteotomy guide plate was generated after fitting it to the lateral surface of the distal humerus using SolidWorks software (Figures 4E,F).

3. Design of the Custom-Made Location Template
  Two guide pins were placed proximal to the POP on the affected humerus and designated as positioning guide pins. A corresponding positioning guide plate was designed using 3D modeling software (SolidWorks) to match the lateral surface of the distal humerus (Figures 4,H).
4. Production Time and Cost

The complete workflow—from CT segmentation and 3D planning to the final printing of the guide set (location, osteotomy, and reduction templates)—required approximately 8–12 h per case, predominantly dedicated to digital simulation and design refinement. The guides were printed using a Formlabs Form 3B+ printer with Surgical Guide resin.

The material cost for each set of patient-specific guides was estimated to be under USD 50. This in-house production model presented a significant cost advantage compared to commercially available custom guide services.

#### Step 4: preoperative simulation

**Figure 5:**
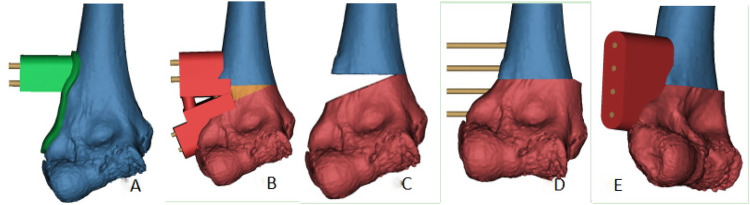
Screen captures showing preoperative simulation the three-dimensional corrective osteotomy with the use of a custom-made osteotomy template combined with location and reduction template. **(A)** location template(green) was placed on the lateral surface of the distal part of the humerus. **(B)** Location template was replaced by a custom-made osteotomy template and firmed fitting with another two pins through guides on osteotomy template. **(C)** The osteotomy was performed through the slits (arrows) on the template and the bone wedge (orange segment) was removed. **(D)** Deformity correction was achieved by bringing the Kirschner wires into parallel status to each other. **(E)** A reduction template was used to maintain the parallel position of the Kirschner wires.

**Figure 6:**
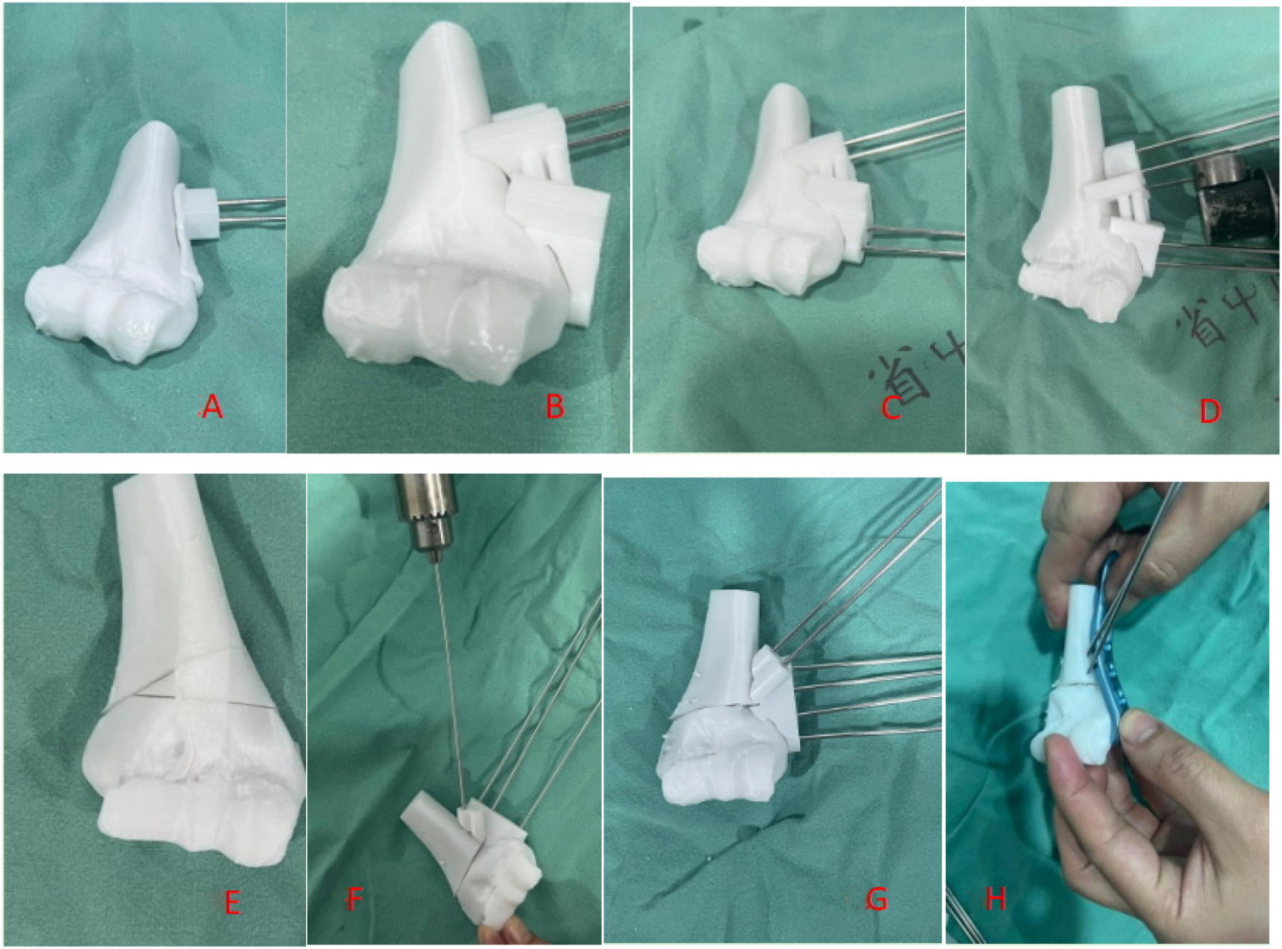
Preoperative simulation the three-dimensional corrective osteotomy with the use of a custom-made osteotomy template combined with location and reduction template in 3D printed model. **(A)** location template was placed on the lateral surface of the distal part of the humerus. **(B,C)** location template was replaced by a custom-made osteotomy template and firmed fitting with another two pins through guides on osteotomy template. **(D)** The osteotomy was performed through the slits on the template. **(E,F)** The bone wedge was removed and deformity correction was achieved by bringing the Kirschner wires into parallel position to each other. **(G)** A reduction template was used to maintain the parallel position of the Kirschner wires and another two Kirschner wires are inserted from the proximal pipes of the template for temporarily fixation. **(H)** internal fixation with lateral Locking Compression Plate (LCP) as preoperatively simulated after removing custom-made surgical osteotomy template. Case A 44 years female presented with a left cubitus varus deformity.

Preoperative simulation the 3D corrective osteotomy was conducted using computer software, incorporating the custom-made osteotomy, location, and reduction templates (Figures 5A–E). A corresponding 3D-printed model was also created to replicate the planned procedure (Figures 6A–H).

#### Step 5: surgical technique

**Figure 7:**
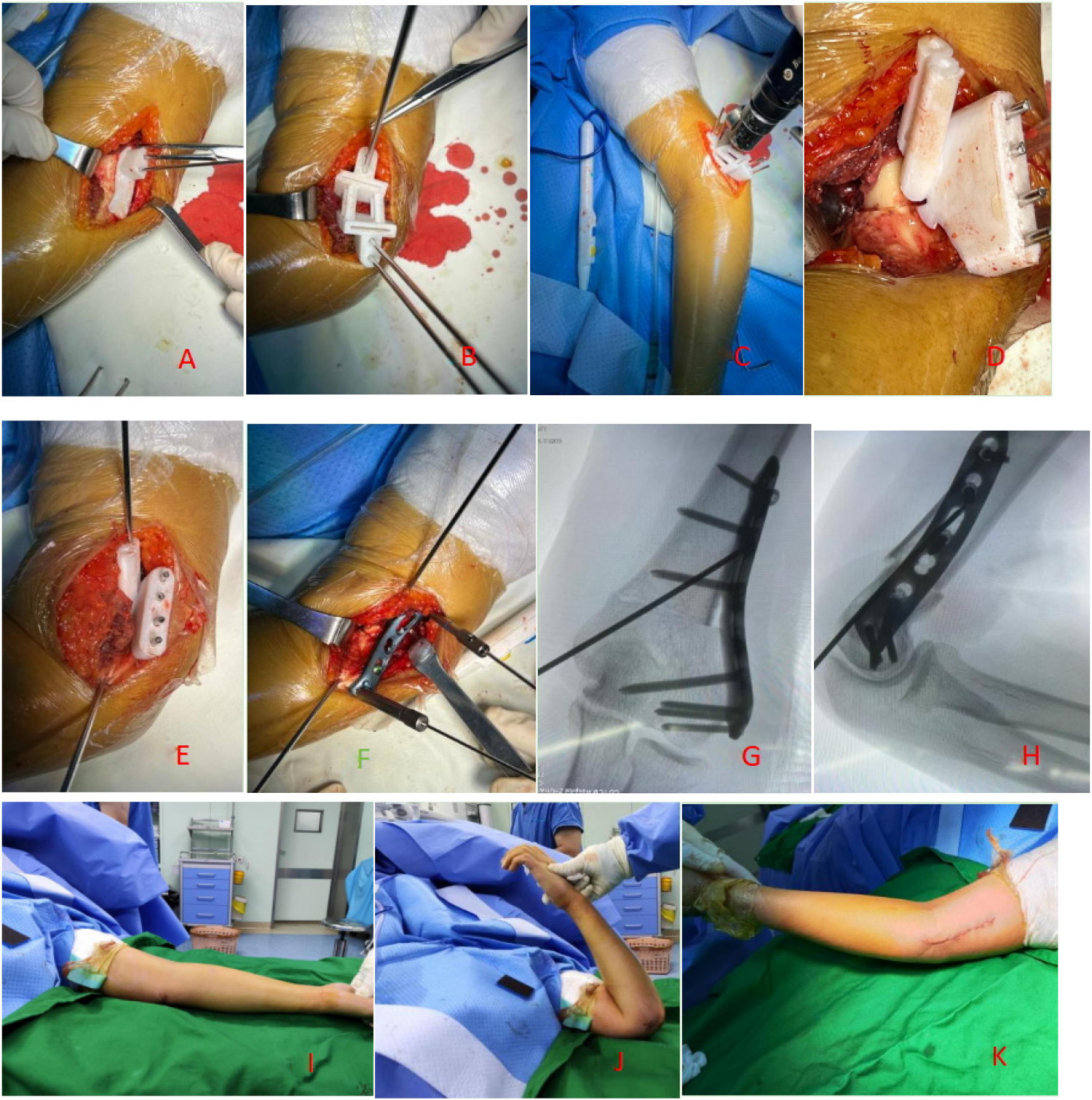
**(A)** Two 2.5 mm kirschner wires were inserted through location template pipes after all edges of the template are in exact contact with lateral surface of the distal humerus. **(B)** location template was replaced by custom-made osteotomy template by two inserted Kirschner wires and firmed fitting with another two Kirschner wires through guides pipes on osteotomy template. **(C)** Accurate osteotomy were completed by a saw through the osteotomy slits on custom-made osteotomy template. **(D,E)** The osteotomy bone wedge was removed and deformity correction was achieved by bringing the Kirschner wires into parallel position and maintain by a reduction template, and then another Kirschner wire are inserted from pipe of the template for temporarily fixation. **(G,H)** AP and lateral view fluoroscopy intraoperative showed the cubitus varus deformity was accurately corrected with internal fixation in good position. **(I,J)** Passive range of-motion intraoperative immediately after surgery was normal and the cubitus varus deformity was accurately corrected. **(K)** A about 8 cm lateral incision was showed in the lateral of elbow.

The patient was placed in the supine position with the affected limb on a side table. Following standard sterilization and draping, a single lateral approach was used. The incision was centered over the lateral epicondyle and extended approximately 8 cm proximally along the lateral supracondylar ridge (Figure 7 K). The skin, subcutaneous tissue, and deep fascia were incised layer by layer. The interval between the triceps and brachioradialis muscles was developed to expose the lateral surface of the distal humerus, with care taken to protect the radial nerve. Subsequently, the pre-designed and sterilized location template was precisely applied to the predetermined area on the lateral surface of the distal humerus. After ensuring all edges of the template were in exact contact with the bone surface, two 2.5 mm Kirschner wires were drilled through the preformed channels in the template to securely fix it to the bone (Figure 7A).

The location template was then removed. Using the two previously inserted Kirschner wires as guides, the custom-made osteotomy template was mounted onto the same position. After confirming proper fit of the template against the bone surface, two additional Kirschner wires were drilled through the remaining channels in the template to further enhance its stability (Figure 7B). Subsequently, using an oscillating saw, osteotomies were performed sequentially along the osteotomy slits on the template to create the proximal osteotomy plane (POP) and the distal osteotomy plane (DOP) (Figure 7C), and the wedge-shaped bone segment was removed.

After the osteotomy, the distal fragment was manually adjusted to the predetermined corrected position. At this point, the reduction template was used to maintain all four Kirschner wires in a parallel alignment, thereby achieving precise three-dimensional reduction (Figure 7D). An additional Kirschner wire was drilled through a specific channel in the reduction template for temporary fixation (Figure 7E). To address the potential risk of ulnar nerve irritation and to provide supplemental medial support, a small additional medial incision, approximately 1 cm in length, was made over the medial epicondyle. Blunt dissection was performed down to the bone surface, and under direct visualization, a Kirschner wire was drilled from the medial epicondyle across the osteotomy site to provide medial support. This maneuver aimed to directly monitor the wire entry point and avoid injury to the ulnar nerve.

For definitive fixation in patients with closed physes, a pre-contoured distal humerus lateral locking compression plate (LCP) was applied through the lateral incision. The plate was not bent intraoperatively. In the skeletally immature patient (Case 4), only Kirschner wires were used for fixation to avoid physeal injury. The K-wire holes in the reduction guide were not designed to correspond to the plate holes; the plate was applied independently after template and lateral wire removal (Figure 7F). After fixation, the medial Kirschner wire was retained, and the lateral temporary fixation wires were removed. Intraoperative C-arm fluoroscopy was used throughout to confirm satisfactory correction angles and proper implant positioning (Figures 7G,H).

Immediate postoperative examination confirmed a normal passive range of motion, and the deformity was satisfactorily corrected through the lateral incision (Figures 7I, K). Active and passive range-of-motion exercises without a splint were initiated on the first postoperative day.

**Figure 8:**
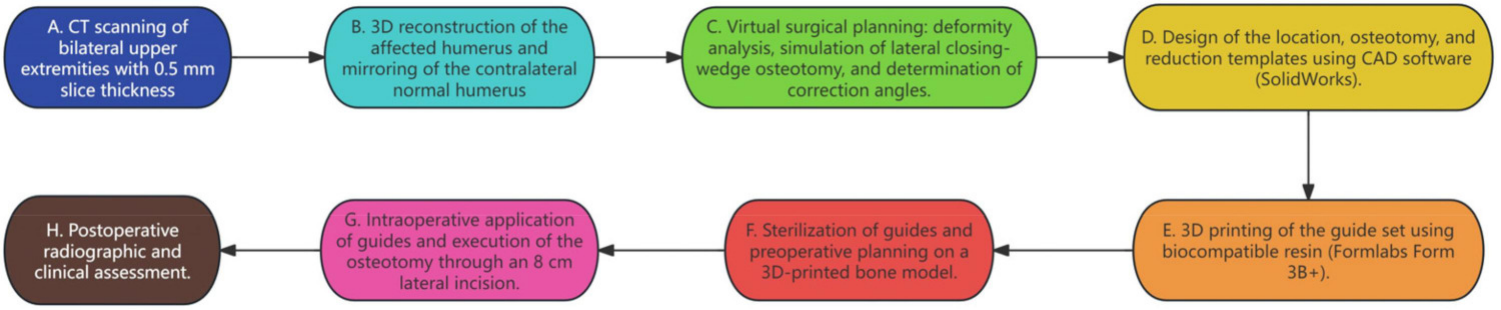
Step-by-step workflow for patient-specific 3D-printed guide production and surgical correction of cubitus varus deformity. The workflow comprises eight main steps: (1) CT acquisition with standardized parameters (0.5 mm slice thickness, low-dose protocol); (2) 3D reconstruction and deformity analysis using Magics RP software, with comparison to mirrored contralateral normal side; (3) virtual osteotomy simulation, including definition of proximal and distal osteotomy planes and calculation of correction angle; (4) design of three custom guides (location, osteotomy, and reduction templates) using SolidWorks software; (5) 3D printing using Formlabs Form 3B+ with Surgical Guide resin; (6) preoperative rehearsal on 3D-printed bone model; (7) surgical execution through 8 cm lateral incision, including guide placement, osteotomy, reduction, and fixation; (8) postoperative assessment and follow-up.

Figure 8 shows the step-by-step workflow for patient-specific 3D-printed guide production and surgical correction of cubitus varus deformity.

### Radiographic evaluation

Standardized anteroposterior (AP) and lateral radiographs of both elbows were obtained preoperatively, immediately postoperatively, and at each follow-up visit. All radiographic measurements were performed independently by two orthopedic surgeons. The carrying angle (CA) was measured on AP radiographs as the angle formed by the intersection of the longitudinal axis of the humeral shaft and the longitudinal axis of the ulnar shaft. A negative value indicated varus alignment. The tilting angle (TA) was measured on lateral radiographs as the angle between the anterior cortical line of the humeral shaft and the axis of the distal metaphysis (the line connecting the centers of the capitellum and trochlea). An increased value typically indicates hyperextension deformity. Internal rotation deformity was assessed clinically by comparing forearm pronation/supination range and shoulder rotation between the affected and contralateral sides, as precise radiographic measurement of rotation was not routinely performed.

### Case presentation

**Figure 9:**
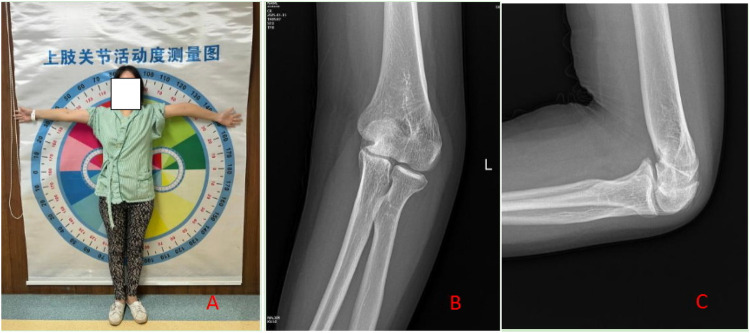
**(A–C)** preoperative photograph and radiographs showed cubitus varus deformity of left elbow.

A 44-year-old female with a 35-year history of left cubitus varus deformity following a childhood supracondylar fracture underwent the aforementioned surgical procedure (Figures 9A–C). Preoperative 3D evaluation revealed a deformity comprising 27° of varus, 0° of extension, and 10° of internal rotation. The preoperative carrying angles were 13.8° (normal side) and −13.4° (affected side), with tilting angles of 52° and 39°, respectively.

The surgical steps were performed as described in the Surgical Technique section. An 8 cm lateral incision was utilized. Intraoperatively, deformity correction was successfully achieved following the guided osteotomy and reduction. For definitive fixation, a lateral locking compression plate (LCP) was applied. Immediate postoperative passive range of motion was normal, confirming accurate correction through the minimally invasive approach (Figures 7I,J).

**Figure 10:**
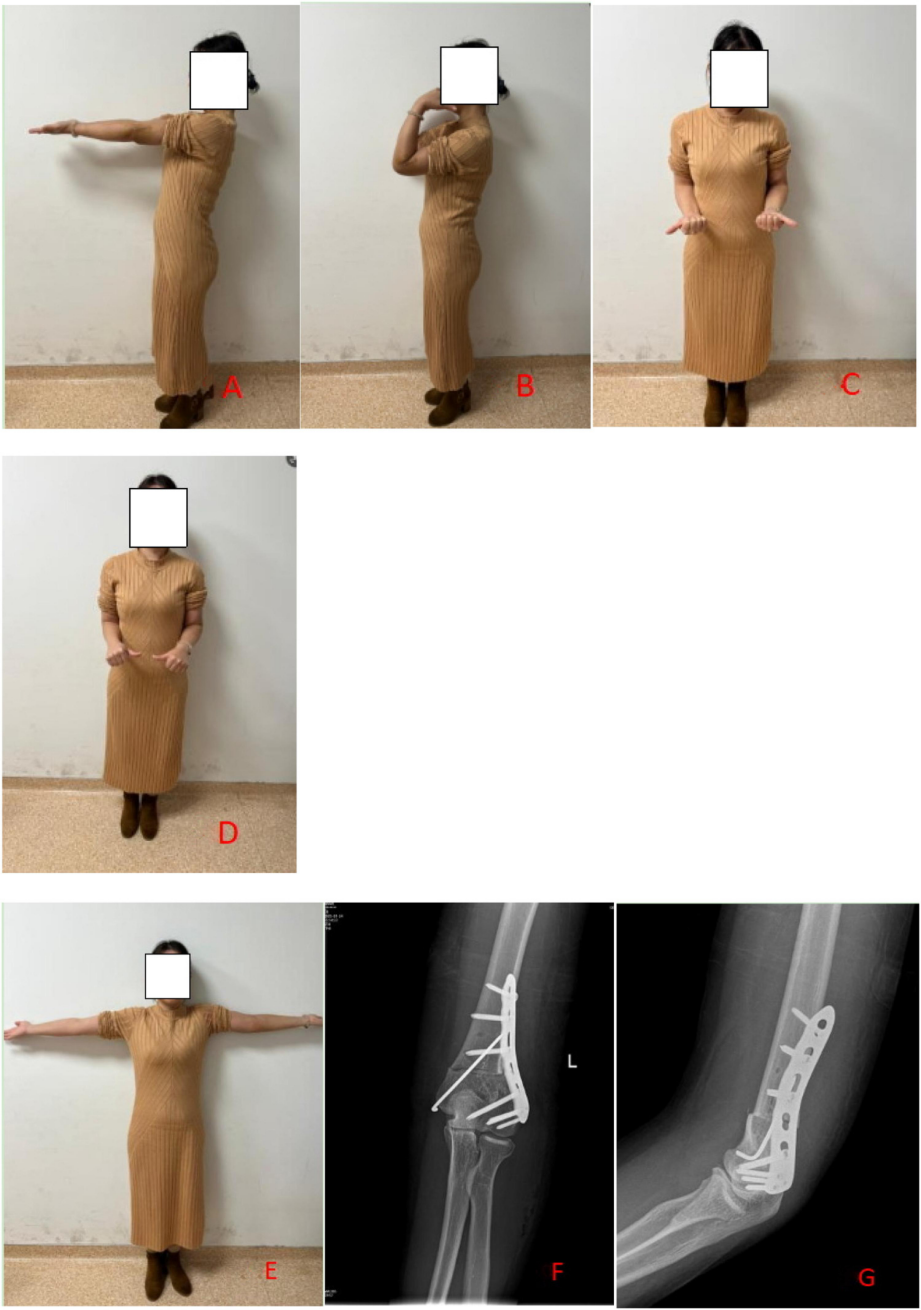
**(A–D)** the left elbow showed excellent function and same with normal right side two weeks postoperation. **(E–G)** Postoperative photograph and radiographs showed cubitus varus deformity of left elbow has been accurate corrected.

At the two-week postoperative follow-up, elbow flexion improved from 120° to 145°, and extension improved from 10° to 0°. Forearm pronation and supination returned to normal (Figures 10A–D). The carrying and tilting angles were corrected to 6.3° and 48°, respectively. Postoperative photographs and radiographs confirmed accurate correction of the left elbow deformity (Figures 10E–I).

## Results

The mean CA and TA on the affected side improved significantly from −15.74° ± 6.58° (varus) and 54.8° ± 7.40°, respectively, before surgery, to 7.77° ± 3.94° (valgus) and to 51.4° ± 2.33°, respectively, after surgery (Table 2). The elbow range of motion returned to normal, with an increase in flexion angle of 24° ± 8° (range: 15°–35°). Hyperextension of the elbow and internal rotation of the shoulder were normalized in all patients. Bone union was achieved at a mean of 2.6 ± 0.49 months (range: 2–3 months) after surgery. Operation time averaged 139.6 ± 22.26 min (range: 116–175 min), and mean intraoperative blood loss was 42 ± 31.87 mL (range: 10–100 mL). The mean planned correction angle was 23.58° ± 8.78° (range: 12.91°–34.63°), and the mean achieved correction angle was 23.51° ± 8.79° (range: 12.43°–33.43°) (Table 3). According to the MEPI (Mayo Elbow Performance Index) assessment, all five patients had excellent outcomes at the final follow-up (mean: 21.6 ± 4.8 months), and none had poor results. One patient (Case 4, 11-year-old male) developed transient ulnar nerve neuropraxia on the first postoperative day, presenting as paresthesia in the ulnar nerve distribution of the hand. The medial Kirschner wire, which had been placed percutaneously, was immediately removed at the bedside. The patient was treated with oral mecobalamin (0.5 mg three times daily) for four weeks. Sensory symptoms fully resolved by the 6-week follow-up, with no residual motor deficits. There were no cases of surgical site infection, implant failure, nonunion, delayed union, loss of correction, or reoperation in this series. No patient reported lateral humeral prominence or dissatisfaction with scar appearance.

**Table 2:** Preoperative and postoperative carrying and tilting angles^a^.

Case	Radiographic parameters (°)			Clinical parameters (°)		
	Carrying angle (deg)			Tiling angle (deg)		
	Preop.	Postop.	Normal side	Preop.	Postop.	Normal side
1	−22.13	12.2	15.29	36	52	52
2	−22.18	11.22	8.43	49	52	52
3	−4.41	8.02	10.21	40	55	53
4	−16.54	1.15	3.07	56	50	50
5	−13.43	6.28	19.71	39	48	53
Mean ± SD	−15.74 ± 6.58	7.77 ± 3.94	10.17 ± 5.72	54.8 ± 7.4	51.4 ± 2.3	52 ± 1.1

a Tilting Angle is defined as the angle between the humeral shaft axis and the line connecting the centers of the capitellum and trochlea on a lateral radiograph.

**Table 3:** Comparison between preoperatively planned correction angles and postoperatively achieved correction angles in five patients undergoing 3D-printed guide-assisted osteotomy for cubitus varus deformity.

Case	Sex/Age (y)	Side	Pre-op CA (°)	Post-op CA (°)	Planned Correction (°)^a^	Achieved Correction (°)^b^	Difference (°)^c^
1	F/12	L	−22.13	12.20	34.63	34.33	0.30
2	M/37	L	−22.18	11.22	32.98	33.40	0.42
3	F/7	R	−4.41	8.02	12.91	12.43	0.48
4	M/11	R	−16.54	1.15	18.14	17.69	0.45
5	F/44	L	−13.43	6.28	19.23	19.71	0.48
Mean ± SD			−15.74 ± 6.58	7.77 ± 3.94	23.58 ± 8.78	23.51 ± 8.79	0.43 ± 0.07

CA, carrying angle; SD, standard deviation.

a Planned correction angle as determined by preoperative 3D simulation (angular dimension of the resected wedge).

b Achieved correction angle calculated as Post-op CA minus Pre-op CA.

c Absolute difference between planned and achieved correction angle.

## Discussion

In adolescents, Cubitus varus deformity is the most common complication following distal humeral fractures (26). Recent studies have demonstrated that cubitus varus is a 3D deformity involving not only varus angulation but also extension and internal rotation of the distal humeral segment (18, 27, 28). As a result, corrective approaches have increasingly focused on 3D techniques to achieve comprehensive improvements in function and appearance (29–33). However, accurately correcting angular deformities in all three planes remains technically challenging for surgeons. It often requires repeated intraoperative adjustments or visual estimation, which may lead to substantial deviations from the desired alignment and suboptimal outcomes (26).

Recent advances in computer simulation technology, along with the use of patient-specific surgical guides and implants created through 3D printing, have addressed many of the challenges associated with correcting cubitus varus deformity. These technologies have enabled accurate, simple, and safe 3D osteotomy procedures (16, 19, 24–26). Several studies have reported excellent outcomes and improved osteotomy accuracy, leading many authors to recommend this technique as an ideal treatment approach for cubitus varus deformity (16, 26, 30, 34, 35).

For example, Jiang et al. (19) reported that the combined rate of excellent and good outcomes was 92.3%. Takeyasu et al. (16) described the use of custom-made osteotomy templates in 30 cases of cubitus varus deformity, with 90% of patients achieving excellent results and the remaining 10% achieving good results. However, compared with conventional procedures, 3D-guided osteotomy often requires a larger surgical field to accommodate the guide. Sri-Utenchai et al. (36) used a standard posterior paratricipital approach with a 20 cm posterior midline incision. Zhang et al. (26) reported the use of double incisions (medial and lateral) in their study. Omori et al. (30) used a lateral approach in 7 cases and a posterior approach in 10 cases, though incision lengths were not specified. Takeyasu et al. 38 also used a lateral approach but did not report incision length. In contrast, our technique achieved excellent functional and radiographic outcomes in all five patients through a single, 8 cm lateral incision.

In this study, we applied a 3D-printed, patient-specific surgical osteotomy template combined with location and reduction guides to perform accurate 3D corrective osteotomy for cubitus varus deformity through a small lateral incision. All patients achieved excellent outcomes, including improved cosmetic appearance, no loss of correction, and no delayed or nonunion at the osteotomy site. Although the 8 cm lateral incision remains visible, all patients expressed satisfaction with the overall cosmetic improvement following deformity correction, and no patient reported concern regarding the scar appearance at final follow-up.

The present study demonstrated excellent accuracy of the 3D-printed patient-specific guide technique, with a mean planned correction angle of 23.58° ± 8.78° and a mean achieved correction angle of 23.51° ± 8.79°, resulting in a mean absolute difference of 0.43° ± 0.07° between planned and achieved correction. This high level of precision—with all deviations under 0.5° from the preoperative plan—demonstrates that the patient-specific guides effectively translated the virtual surgical plan into intraoperative reality with minimal error.

This level of accuracy is consistent with previous reports on 3D-guided osteotomies for cubitus varus deformity. Omori et al. (30) evaluated the accuracy of 3D corrective osteotomy using custom-made surgical guides in 17 patients and reported mean errors of 0.6° ± 0.7° in varus-valgus rotation, 0.8° ± 1.3° in flexion-extension rotation, and 2.9° ± 2.8° in internal-external rotation. The varus-valgus correction accuracy in our series (mean absolute difference 0.43° ± 0.07°) is comparable to or slightly better than that reported by Omori et al., with all our deviations falling within one standard deviation of their reported mean error.

Zhang et al. (26) compared 3D printing-assisted osteotomy with conventional techniques in adolescent patients with cubitus varus deformity and found that the 3D printing group had significant advantages in operation time, intraoperative blood loss, rate of excellent correction, and parental satisfaction with appearance (*P* < 0.001, *P* < 0.001, *P* = 0.019, and *P* = 0.023, respectively). However, they reported no significant difference in postoperative carrying angle between the 3D printing and conventional groups (*P* = 0.626). Our study did not include a conventional control group for direct comparison, but the high accuracy achieved (0.43° ± 0.07° deviation) suggests that our technique—combining location, osteotomy, and reduction templates—may offer improved precision compared to conventional methods, while the short operative time (139.6 ± 22.3 min) and minimal blood loss (42 ± 31.9 mL) in our series align with the advantages of 3D-guided techniques reported by Zhang et al.

Our series included both pediatric and adult patients (age range: 7–44 years), demonstrating the adaptability of this patient-specific 3D-guided technique. The fundamental planning principles and guide design are independent of skeletal maturity. The primary distinction lies in the choice of final fixation: locking plate fixation for skeletally mature patients provides stable construct for early mobilization, while K-wire fixation in the skeletally immature patient avoids physeal injury while providing sufficient stability for healing. This flexibility underscores the method's broad utility in correcting cubitus varus deformities regardless of patient age.

There are several reasons why a single, limited lateral incision was sufficient to achieve accurate 3D correction in our cases. First, we used a conventional closed lateral wedge osteotomy. An 8 cm lateral incision was sufficient to allow the osteotomy template to be properly attached to the lateral surface of the distal humerus. Second, although our osteotomy template was relatively larger than those used in some previous studies (23, 26, 37, 38), we designed and added a location template that was smaller than the main guide. Two location Kirschner wires were accurately inserted using this smaller guide through the 8 cm incision. Afterward, the smaller guide was replaced with the larger osteotomy template without requiring additional incision length. This method ensured a secure fit and prevented loosening or shifting of the guide during osteotomy, thereby improving surgical precision. Third, the reduction guide was specifically designed to assist with post-osteotomy alignment by temporarily holding the four Kirschner wires in a parallel position. This not only ensured an accurate reduction without extending the original incision but also significantly shortened the reduction time. Using this technique, all five patients achieved single-attempt osteotomy success. Postoperative appearance and correction angles matched the preoperative plan and bone healing was confirmed at the osteotomy site approximately three months after surgery.

However, Several practical considerations should be noted when applying this technique clinically. First, the cutting slits on the osteotomy template were prone to breakage due to oscillation of the bone saw during the procedure, particularly when the slit thickness was insufficient. To address this issue, we increased the slit thickness to at least 3 mm. This design choice prioritized guide robustness to prevent fracture during the osteotomy, particularly the second cut. While a wider slit theoretically allows more saw blade movement, accuracy was maintained through secure guide fixation to the bone and precise surgical technique, with postoperative measurements confirming close alignment to the preoperative plan. We also prepared two surgical guides per case, both of which were sterilized prior to surgery. One patient developed ulnar nerve paralysis postoperatively due to the placement of the medial Kirschner wire. The nerve function fully recovered after immediate wire removal and four weeks of treatment with neurotrophic agents. Following this event, we began using a 1 cm incision over the medial epicondyle to directly expose the Kirschner wire entry point, and no further ulnar nerve injuries were observed. Although previous reports have described limitations of 3D-printed guide plates, such as a risk of correction loss due to reduced bone contact area and compromised fixation stability (20, 31, 39–41), our study did not observe these issues.

There were no cases of correction loss, delayed union, nonunion, or fixation failure in our study. This may be attributed to the stable construct achieved with primary fixation using a lateral locking compression plate (LCP), supplemented by a medial Kirschner wire. The LCP provided the main rigidity required for early active functional exercise, while the medial wire acted as an auxiliary stabilizer to counter varus stress and enhance rotational control. Radiation exposure from CT imaging is another concern (24, 29, 32), but this can be minimized through low-dose imaging protocols and improved image-processing technologies.

A commonly cited disadvantage of 3D printing is the requirement for additional equipment and software for simulation and model generation, which can pose a technical barrier. Building upon this point, we acknowledge that the digital planning and guide design process itself requires proficiency in specialized 3D modeling software, which may present a learning curve for surgical teams without dedicated engineering support. However, it is crucial to recognize that the core strength and innovation of this approach lie in its conceptual framework—the execution of a precise, preoperatively simulated correction—rather than solely in the in-house manufacturing of the guides. For clinical centers lacking the technical resources or inclination to handle design and printing internally, the preoperative planning data (the virtual correction plan) can be effectively transferred to commercial medical modeling companies for guide production, a service increasingly offered globally. Therefore, the primary clinical adoption barrier is not the physical manufacturing, but rather the commitment to obtaining preoperative CT imaging and investing time in detailed virtual planning. In our experience, having the team handle all design and printing in-house significantly reduced production time and cost, but the commercial outsourcing pathway remains a viable and practical alternative to achieve the same surgical goal. We believe the investment in preoperative planning is justified by the resultant gains in surgical accuracy, reproducibility, and potentially reduced operative time. Previous studies have also suggested that patient-specific guides may reduce the risk of revision surgery by lowering complication rates and overall medical expenses.

Finally, this study has several limitations, including its retrospective, non-randomized design, small sample size of only five patients, and relatively short follow-up period, all of which limit the generalizability of the findings and may introduce subjective bias. Second, the follow-up period is variable and relatively short (range: 6–35 months), with one patient having only 6 months of follow-up. While no early loss of correction was observed, longer-term follow-up is essential to monitor for late recurrence or hardware-related issues. Third, the lack of a control group undergoing conventional osteotomy means that our comparisons to other techniques are indirect and should be interpreted cautiously. Finally, all procedures were performed by the same senior surgical team to ensure consistency in this initial technical description. However, the reproducibility of this technique in less specialized centers remains to be demonstrated. Despite these limitations, we believe this study provides valuable preliminary evidence for the feasibility and precision of this 3D-guided approach. The consistent precision and safety observed in our consecutive series align with and reinforce the growing body of literature advocating for 3D-guided osteotomy as a reliable standard for managing this complex three-dimensional deformity.

## Conclusion

This study demonstrates that 3D-printed, patient-specific surgical osteotomy guides—combined with location and reduction templates—enable accurate 3D correction of cubitus varus deformity through a limited lateral incision. Based on computer simulation, this surgical technique is reproducible and represents a promising therapeutic option. However, larger comparative studies with longer follow-up are necessary to fully establish its benefits relative to conventional techniques.

## Data Availability

The raw data supporting the conclusions of this article will be made available by the authors, without undue reservation.
